# Cultural comparison of positive development: network analyses of adolescents’ ecological systems in Mainland and non-Mainland China

**DOI:** 10.1186/s40359-026-05106-z

**Published:** 2026-07-07

**Authors:** Xiaoran Xue, Xiaofang Weng, Mengyu Miranda Gao, Wanting Yang, Rongqi Ding, Wei Cui, Zhuo Rachel Han

**Affiliations:** 1 Beijing Key Laboratory of Applied Experimental Psychology, National Demonstration Center for Experimental Psychology Education, Faculty of Psychology, Beijing Normal University, Beijing, China; 2 Faculty of Psychology, Shandong Normal University, Jinan, China

**Keywords:** Adolescents’ positive development, Ecological systems, Subcultural context, Network analysis

## Abstract

Understanding the ecological contexts of adolescents’ positive development across different sociocultural settings remains critical but underexamined. This study examined how family and school factors, along with adolescents’ positive developmental outcomes, interrelate as an ecological network across subcultural groups in Greater China. Network analyses revealed a more tightly connected configuration within the Mainland China (MC) group, with denser connections among family factors, school factors, and positive developmental outcomes. In contrast, the non-Mainland China (non-MC) network exhibited weaker overall connectivity. Reading competence and resilience were the most central outcomes within the MC network, whereas parental emotional support demonstrated greater bridging strength within the non-MC network. Additionally, belongingness emerged as a central factor across both groups. Findings highlight cross-subcultural differences in adolescents’ developmental ecologies and provide a foundation for culturally responsive interventions.

**Supplementary Information:**

The online version contains supplementary material available at https://doi.org/10.1186/s40359-026-05106-z.

## Introduction

Understanding adolescents’ development requires a multi-system perspective. Bronfenbrenner’s Ecological Systems Theory emphasizes multiple layers of influence, ranging from family and school to broader societal contexts [4]. Cultural and educational contexts modulate how these systems function, resulting in different developmental patterns. However, within a shared Chinese cultural heritage, the ways in which subcultural contexts shape the interrelations between family–school factors and indicators of positive development remain underexplored.

Within Greater China, MC and the non-MC regions (Hong Kong, Macao, and Taiwan) share historical roots but differ in educational philosophies and cultural orientations. MC is relatively more examination-driven, whereas the non-MC regions place a more balanced emphasis on academic and socioemotional goals and maintain clearer boundaries between family and school [16, 54, 58]. These subcultural orientations are associated with distinct patterns of interrelations between family and school factors. Yet how these interrelations differ across regions of Greater China remains underexplored, motivating a focus on their structural organization rather than on mean-level differences. A network perspective enables comparisons of global strength, node strength, bridge strength, and bridge expected influence, clarifying whether family factors, school factors, and positive developmental outcomes are more tightly integrated or more functionally differentiated across subcultural contexts. Using data from the 2018 Programme for International Student Assessment (PISA), this study applies network analysis to compare MC and non-MC family–school networks and to examine how subcultural contexts are associated with adolescents’ positive development.

### The role of different systems in adolescents’ positive development

Adolescents’ development occurs within interconnected systems that can both facilitate and constrain growth. Family and school serve as key immediate contexts, and their interrelations form part of the mesosystem through which adolescents receive socialization [4]. In the developmental assets framework, these contextual resources are conceptualized as external assets that foster positive development [2, 17, 27]. Positive development is generally conceptualized as a multidimensional construct that includes competencies, strengths, adaptive functioning, and well-being [41]. In the present study, adolescents’ positive development was represented by three theoretically distinct domains: reading competence, resilience, and positive feelings. Reading competence reflects achievement-related functioning; resilience captures adaptive capacity in response to stress and environmental demands; and positive feelings represent adolescents’ positive subjective experiences. Together, these indicators capture complementary facets of positive development, spanning achievement-related functioning, adaptive capacity, and subjective well-being.

#### Family system

Family shapes adolescent development through both material resources and psychological resources. Socioeconomic status (SES), as a material resource, reflects access to financial resources, education, and opportunities. Adolescents from lower-SES backgrounds often face resource scarcity, which is associated with greater vulnerability to emotional and psychological distress when facing challenges [1, 50]. Limited resources may also restrict access to quality education. In contrast, adolescents from higher-SES families tend to have greater access to private tutoring, higher-quality schools, and culturally valued learning opportunities, all of which are associated with better academic performance [6, 7]. Beyond material resources, emotional support serves as a crucial psychological resource essential for adolescents’ positive development, promoting mental health, well-being, resilience, and academic performance [5, 31, 50]. In contrast, feelings of loneliness and isolation within the family can undermine adolescents’ mental health, as reflected in reduced resilience and an increased risk of psychological problems [45].

#### School system

Peer relationships and school climate play pivotal roles in adolescents’ positive development. School belongingness is a well-established protective factor associated with better academic performance [8] as well as higher positive feelings and life satisfaction [50]. In contrast, being bullied is associated with lower subjective well-being, lower positive feelings, poorer academic performance, and higher negative affect [20, 50, 55, 57]. Cooperative school environments generally promote subjective well-being, supportive peer relationships, and academic performance [29, 39, 50]. Competitive environments have more complex implications: competition may be associated with enhanced academic performance, but it may also coincide with greater mental health challenges, particularly in highly ranking-oriented settings [21, 49].

Adolescents’ development can also be understood through the interplay between risk and protective factors embedded in family and school systems. Risk factors generally refer to conditions that may hinder adolescents from achieving developmental expectations, such as low SES, experiences of bullying, or highly competitive academic environments. Such risk factors can increase the likelihood of adverse developmental outcomes by constraining adolescents’ resources or weakening their support systems. The developmental implications of these risks may also depend on the presence of protective factors. Protective factors—such as family emotional support, cooperative school climates, and school belongingness—can buffer adolescents against contextual risks and provide psychological resources for adaptive responses to environmental challenges [30, 33].

### Adolescents’ positive development in Chinese subcultures

Chinese cultural contexts are internally diverse, and this diversity provides an important basis for examining adolescents’ positive development beyond broad East–West comparisons. Previous studies have predominantly examined adolescents’ development through broad East–West comparisons, often emphasizing individualistic values [14]. Although such comparisons are valuable, variations within Eastern cultural contexts, particularly among subcultures within a shared Chinese cultural heritage, remain underexplored. Chinese culture, rooted in Confucian traditions, places strong emphasis on family hierarchy, discipline, and collective responsibility [14, 26]. At the same time, family practices, educational systems, and developmental expectations vary across Chinese subcultural contexts, making it necessary to examine how family and school systems are linked with adolescents’ positive development within Greater China.

Within the broader Chinese context, significant contextual differences emerge between MC and the non-MC regions. In MC, the education system strongly emphasizes examination performance and academic achievement; parents typically hold high academic expectations, and many higher-SES families invest heavily in educational resources to enhance academic outcomes [58]. Parenting practices are also evolving, with mothers in Beijing showing higher autonomy support than their counterparts in Hong Kong in prior research [14]. Shadow education—paid after-school tutoring provided by private companies or individual tutors outside the formal school system—is widespread in MC and is often exam-oriented [58]. In contrast, the non-MC regions feature more diverse educational pathways and relatively flexible examination systems. Although Hong Kong, Macao, and Taiwan differ in governance arrangements and educational histories, they share several systemic characteristics, such as diversified educational pathways, greater institutional flexibility, and more differentiated family–school roles. In these contexts, shadow education may be more closely linked to personal growth and enrichment rather than to examination preparation alone [53], and such involvement has been associated with stronger academic performance, better emotional well-being, greater school belongingness, and a heightened sense of purpose [9, 26]. Prior evidence from Taiwan also suggests that supportive family–school dynamics are associated with adolescents’ psychological resilience, including among adolescents from single-parent families [19]. Family, school, and educational practices provide important pathways through which cultural contexts are linked with adolescents’ development.

### The present study

This study examines how family factors, school factors, and adolescents’ positive development are organized within MC and non-MC ecological networks, shifting the focus from traditional East–West comparisons to subcultural variations within the Chinese cultural context. MC refers to the four Mainland Chinese regions available in PISA 2018—Beijing, Shanghai, Jiangsu, and Zhejiang—whereas non-MC refers to Hong Kong, Macao, and Taiwan. This design allows us to examine how adolescents’ positive development is organized within different family–school configurations under a shared Chinese cultural heritage.

The ecological-systems perspective requires an analytical approach capable of representing multiple family, school, and positive developmental indicators within the same structure. Network analysis provides such an approach by estimating conditional associations among variables and summarizing the network at the system, node, and edge levels. At the system level, global strength captures the overall tightness of the ecological network; at the node level, node strength captures a node’s overall connectivity, whereas bridge strength and bridge expected influence capture a node’s cross-system connectivity and help identify potential linkage points across systems; and at the edge level, edge weights describe specific conditional associations after accounting for other variables in the network. Beyond mean-level comparisons, these indices characterize how family and school factors are structurally connected with positive developmental outcomes within each subcultural context. In this study, variables within family systems (i.e., SES and parental emotional support), school systems (i.e., being bullied, belongingness, cooperation, and competition), and positive developmental outcomes (i.e., reading competence, resilience, and positive feelings) were modeled as nodes, with edges representing partial correlations that quantify unique associations among these variables. We used the Network Comparison Test (NCT) to compare global strength, node strength, bridge strength, bridge expected influence, and edge weights across groups [11, 22, 47]. These indices correspond to the three research questions concerning network tightness, the structural roles of positive developmental outcomes, and the relative connections between family and school systems.

The first research question is whether the networks of MC and non-MC adolescents differ in structural tightness, operationalized as global network strength. Prior research suggests that MC contexts place strong emphasis on academic achievement and coordinated family–school efforts [43, 58]. In contrast, non-MC contexts may involve more flexible educational structures and more differentiated family–school roles [42, 53]. These contextual differences suggest that family and school resources may be more tightly coordinated in MC contexts, forming a more interconnected developmental ecology. This study proposes that the global strength of the MC network exceeds that of the non-MC network (*H1*).

The second research question is whether reading competence, resilience, and positive feelings differ in structural position and cross-system linkage between MC and non-MC networks. Evidence indicates that examination-oriented MC contexts may make reading competence more closely embedded in adolescents’ developmental ecology [43, 58], whereas non-MC contexts may place a more balanced emphasis on academic success and socioemotional well-being [26, 42]. These differences suggest that positive developmental outcomes may serve different functions within MC and non-MC developmental systems, leading them to occupy different structural positions in the network. Among these outcomes, resilience may be particularly sensitive to contextual variation because it reflects adolescents’ capacity to adapt to environmental demands. As a critical ecological resource, resilience buffers against stress and supports adaptation, with its function shaped by the cultural context [32, 46]. In MC contexts, resilience may be more closely tied to academic adaptation under pressure [25], whereas in non-MC contexts, it may be more closely aligned with positive feelings and social support. This study proposes that, compared with the non-MC network, reading competence will show higher node strength and higher bridge strength in the MC network, while resilience will show higher bridge strength and greater bridge expected influence in the MC network. In contrast, positive feelings are expected to show higher bridge expected influence in the non-MC network (*H2*).

The third research question is whether school and family systems differ in their relative connections with adolescents’ positive development across MC and non-MC networks. In MC, where academic success is highly prioritized, school-related factors are expected to be more strongly connected with adolescent development than family-related factors [49]. In contrast, in non-MC contexts, family support and interpersonal relationships may be more closely connected with adolescents’ socioemotional well-being [14, 51]. These patterns suggest that family- and school-related factors may differ in their relative positions within MC and non-MC developmental systems. This study proposes that school-related factors show stronger connections with resilience and reading competence in the MC network, whereas family-related factors show stronger connections with positive feelings and resilience in the non-MC network (*H3*).

## Methods

### Participants

The data used in this study were drawn from PISA 2018. PISA, the OECD’s Programme for International Student Assessment, is a triennial assessment of 15-year-old students’ ability to apply their knowledge and skills in reading, mathematics, and science to meet real-world challenges. Beyond literacy in reading, mathematics, and science, PISA also assesses broader competencies, including collaborative problem solving, financial literacy, and global competence, as well as developmental indicators such as well-being.

PISA 2018 used a two-stage stratified sampling design, in which schools were sampled first and students were then sampled within those schools. Data from MC and non-MC regions were selected for analysis. For the MC group, data were drawn from the four MC regions available in PISA 2018: Beijing, Shanghai, Jiangsu, and Zhejiang. This sample included 12,058 students from 361 schools, with ages ranging from 15.33 to 16.25 years (*M* = 15.78, *SD* = 0.30); 47.89% were girls. For the non-MC group, data were drawn from Hong Kong, Macao, and Taiwan in the PISA 2018 database. This sample included 17,055 students from 370 schools, with ages ranging from 15.25 to 16.33 years (*M* = 15.77, *SD* = 0.29); 49.49% were girls.

### Measures

The variables used in this study were derived from the PISA 2018 student questionnaire. Three positive development outcomes were included: reading competence, resilience, and positive feelings. Contextual indicators represented family factors (SES and emotional support) and school factors (being bullied, belongingness, cooperation, and competition). See Table 1 for detailed descriptions of the measures.

**Table 1:** Descriptive statistics for positive developmental outcomes and ecological factors among MC and non-MC adolescents

Variable	MC (47.89% female) *n* = 12,058		non-MC (49.49% female) *n* = 17,055		Description
	M	SD	M	SD	
Age	15.78	0.30	15.77	0.29	
Reading competence	0.08	0.87	−0.32	0.93	3 items (e.g., I am able to understand difficult texts.)
Resilience	−0.08	0.96	−0.26	0.90	5 items (e.g., When I’m in a difficult situation, I can usually find my way out of it.)
Positive feelings	0.10	0.89	0.01	1.00	3 items (e.g., How often do you feel as described below? Happy)
Family: emotional support	0.01	0.93	0.01	0.91	3 items (e.g., My parents support me when I am facing difficulties at school.)
Family: SES	−0.36	1.09	−0.44	0.96	A composite score based on three indicators: highest parental occupation, parental education, and home possessions
School: cooperation	0.23	1.01	0.15	0.97	3 items (e.g., It seems that students are cooperating with each other.)
School: competition	0.42	0.82	0.21	0.84	3 items (e.g., Students seem to share the feeling that competing with each other is important.)
School: being bullied	−0.23	0.88	−0.09	0.97	3 items (e.g., Other students made fun of me.)
School: belongingness	−0.15	0.91	−0.25	0.81	6 items (e.g., Thinking about your school: I make friends easily at school.)

*M* Mean, *SD* Standard deviation, *SES* Socioeconomic status

#### Reading competence

Reading competence was measured using three self-concept items: (a) I am a good reader; (b) I am able to understand difficult texts; and (c) I read fluently. Adolescents rated each item on a four-point scale (1 = *strongly disagree*, 2 = *disagree*, 3 = *agree*, 4 = *strongly agree*). Higher scores indicate greater reading competence. Cronbach’s alpha coefficients were 0.79 for the MC group and 0.82 for the non-MC group, respectively.

#### Resilience

Resilience was assessed using five items: (a) I usually manage one way or another; (b) I feel proud that I have accomplished things; (c) I feel that I can handle many things at a time; (d) My belief in myself gets me through hard times; and (e) When I am in a difficult situation, I can usually find my way out of it. Adolescents rated each item on a four-point scale (1 = *strongly disagree*, 2 = *disagree*, 3 = *agree*, 4 = *strongly agree*). Higher scores indicate greater resilience. Cronbach’s alpha coefficients were 0.80 for the MC group and 0.78 for the non-MC group, respectively.

#### Positive feelings

Positive feelings were assessed using three items based on the following statements: Thinking about yourself and how you normally feel, how often do you feel (a) joyful, (b) cheerful, and (c) happy? Adolescents rated each item on a four-point scale (1 = *never*, 2 = *rarely*, 3 = *sometimes*, 4 = *always*). Higher scores indicate greater positive feelings. Cronbach’s alpha coefficients were 0.79 for the MC group and 0.87 for the non-MC group, respectively.

#### Family socioeconomic status

Family socioeconomic status was indicated by the PISA index of economic, social, and cultural status (SES). This index is derived from three variables: parents’ highest level of education, parents’ highest occupational status, and home possessions, including books. Parents’ education levels were categorized according to the International Standard Classification of Education categories ranging from 0 to 6, based on students’ responses regarding their parents’ education. The highest educational level of either parent was used to estimate years of schooling. Occupational data were coded using four-digit International Standard Classification of Occupations codes and mapped to the international socio-economic index of occupational status. Three indices were calculated: the father’s occupational status, the mother’s occupational status, and the highest occupational status of the parents. Students also reported on 16 household items and the number of possessions and books at home. Home possessions constitute a composite index of household items. Values for students with missing information on these variables were imputed using regression. SES was computed by equally weighting the standardized components. The final SES variable is standardized, with 0 representing the average OECD student and 1 representing one standard deviation across equally weighted OECD countries.

#### Family emotional support

Adolescents reported their perceived parental emotional support using three items: (a) My parents support my educational efforts and achievements; (b) My parents support me when I am facing difficulties at school; and (c) My parents encourage me to be confident. These items were designed to measure parental emotional support, rather than instrumental support (e.g., hours spent supervising homework). Adolescents rated each item on a four-point scale (1 = *strongly disagree*, 2 = *disagree*, 3 = *agree*, 4 = *strongly agree*), with higher scores indicating greater levels of parental emotional support. Cronbach’s alpha coefficients were 0.91 for the MC group and 0.88 for the non-MC group, respectively.

#### Being bullied

Three items were used to assess being bullied: During the past 12 months, how often have you had the following experiences in school: (a) Other students deliberately marginalized me; (b) Other students made fun of me; and (c) I was threatened by other students. Adolescents rated each item on a four-point scale (1 = *never or almost never*, 2 = *a few times a year*, 3 = *a few times a month*, 4 = *once a week or more*). Higher scores indicate a greater frequency of being bullied. Cronbach’s alpha coefficients were 0.74 for the MC group and 0.72 for the non-MC group, respectively.

#### Belongingness

Six items were used to assess school belongingness: Thinking about your school: to what extent do you agree with the following statements: (a) I feel like an outsider (or marginalized) at school; (b) I make friends easily at school; (c) I feel like I belong at school; (d) I feel awkward and out of place in my school; (e) Other students seem to like me; and (f) I feel lonely at school. Adolescents rated each item on a four-point scale (1 = *strongly disagree*, 2 = *disagree*, 3 = *agree*, 4 = *strongly agree*). Items (a), (d), and (f) were reverse-scored. Higher scores indicate greater belongingness at school. Cronbach’s alpha coefficients were 0.83 for the MC group and 0.80 for the non-MC group, respectively.

#### School cooperation

Three items were used to assess the academic climate of cooperation within the school. Students indicated how true the following statements were: (a) Students seem to value cooperation; (b) It seems that students are cooperating with each other; and (c) Students seem to share the feeling that cooperating with each other is important. Adolescents rated each item on a four-point scale (1 = *not at all true*, 2 = *slightly true*, 3 = *very true*, 4 = *extremely true*). Higher scores indicate a greater level of school cooperation. Cronbach’s alpha coefficients were 0.93 for the MC group and 0.94 for the non-MC group, respectively.

#### School competition

Three items were used to assess the academic climate of competition within the school. Students indicated how true the following statements were: (a) Students seem to value competition; (b) It seems that students are competing with each other; and (c) Students seem to share the feeling that competing with each other is important. Adolescents rated each item on a four-point scale (1 = *not at all true*, 2 = *slightly true*, 3 = *very true*, 4 = *extremely true*). Higher scores indicate greater school competition. Cronbach’s alpha coefficients were 0.84 for the MC group and 0.90 for the non-MC group, respectively.

### Data analysis

All statistical analyses were conducted in R (Version 4.3.2). For preliminary analyses, descriptive statistics and correlations among the study variables were computed for MC and non-MC adolescents. Subsequently, the levels of family and school factors, as well as each positive developmental outcome, were compared between MC and non-MC adolescents using *t*-tests. To assess the potential influence of PISA’s school-based sampling structure, intraclass correlation coefficients (ICCs) were calculated for all study variables and are reported in Supplementary Table S1. The primary networks were estimated using observed student-level data; sampling weights and school-level clustering adjustments were not applied.

Missing data were minimal across study variables (see Supplementary Table S2), ranging from 0.56% to 1.34% in the MC sample and from 1.63% to 4.15% in the non-MC sample. Given the low proportion of missingness, pairwise deletion was employed during network estimation. To examine potential systematic missingness, participants with any missing data were compared with those with complete data on demographic, developmental, and contextual variables (Supplementary Table S3). To assess the robustness of the findings, sensitivity analyses were conducted by re-estimating all networks using complete-case data.

To examine measurement comparability across MC and non-MC groups, multi-group confirmatory factor analyses were conducted on the multi-item constructs included in the network. Results for configural and metric invariance are reported in Supplementary Table S4.

For the network analyses, separate networks were estimated for MC and non-MC adolescents, incorporating factors from family systems, school systems, and positive developmental outcomes. Nodes represented the study variables, whereas edges represented regularized partial correlations between pairs of nodes after controlling for all other nodes in the network. Node strength was calculated as the sum of the absolute values of the edges connected to a node, indicating the overall connectivity of that node within the network. Bridge strength was calculated as the sum of the absolute values of edges connecting a node to nodes in other communities, and bridge expected influence was calculated as the sum of signed cross-community edge weights [22]. In this study, communities were defined as family factors, school factors, and positive developmental outcomes. Edge weights were used to identify the strongest unique associations among family system variables, school system variables, and positive developmental outcomes within each network.

A series of R packages were applied, including “qgraph” for network visualization [10], “glasso” for network estimation [13], and “bootnet” for network stability assessment [11]. Within-network differences in node strength were also computed for each network.

We further applied the “NetworkComparisonTest (NCT)” package to compare network structures between MC and non-MC adolescents [47]. Specifically, we tested the invariance of the overall network structure and global strength, and examined between-network differences in node strength, bridge strength, bridge expected influence, and edge weights. To assess the stability of the estimates, we performed 1,000 bootstrap iterations and examined the stability of node and edge estimates across bootstrap samples [40]. Global strength was interpreted based on the direction and statistical significance of the NCT, with higher values indicating greater overall network connectivity [47]. Bridge strength and bridge expected influence were interpreted based on the relative ranking of nodes within each network and their between-network differences. Higher bridge strength values indicate stronger overall cross-system connectivity, whereas bridge expected influence reflects the signed direction and magnitude of cross-system connections [22]. Specific connections were interpreted based on the sign, magnitude, and Bonferroni-corrected significance of edge weights, with larger absolute edge weights indicating stronger unique associations between nodes after controlling for all other nodes in the network.

To evaluate the appropriateness of grouping Hong Kong, Macao, and Taiwan into a single non-MC category, additional descriptive and network comparison analyses were conducted. Descriptive statistics for each subregion are presented in Supplementary Table S5. Pairwise Network Comparison Tests examining differences in network structure, global strength, bridge strength, bridge expected influence, and edge weights among the three subregions are reported in Supplementary Table S6.

This study was not preregistered.

## Results

### Preliminary results

Descriptive statistics for all study variables are presented in Table 1. Independent-samples *t*-tests indicated that all study variables listed in Table 1 showed statistically significant differences between MC and non-MC adolescents (*p*s < .001).

### Network estimation and network comparison

**Fig. 1:**
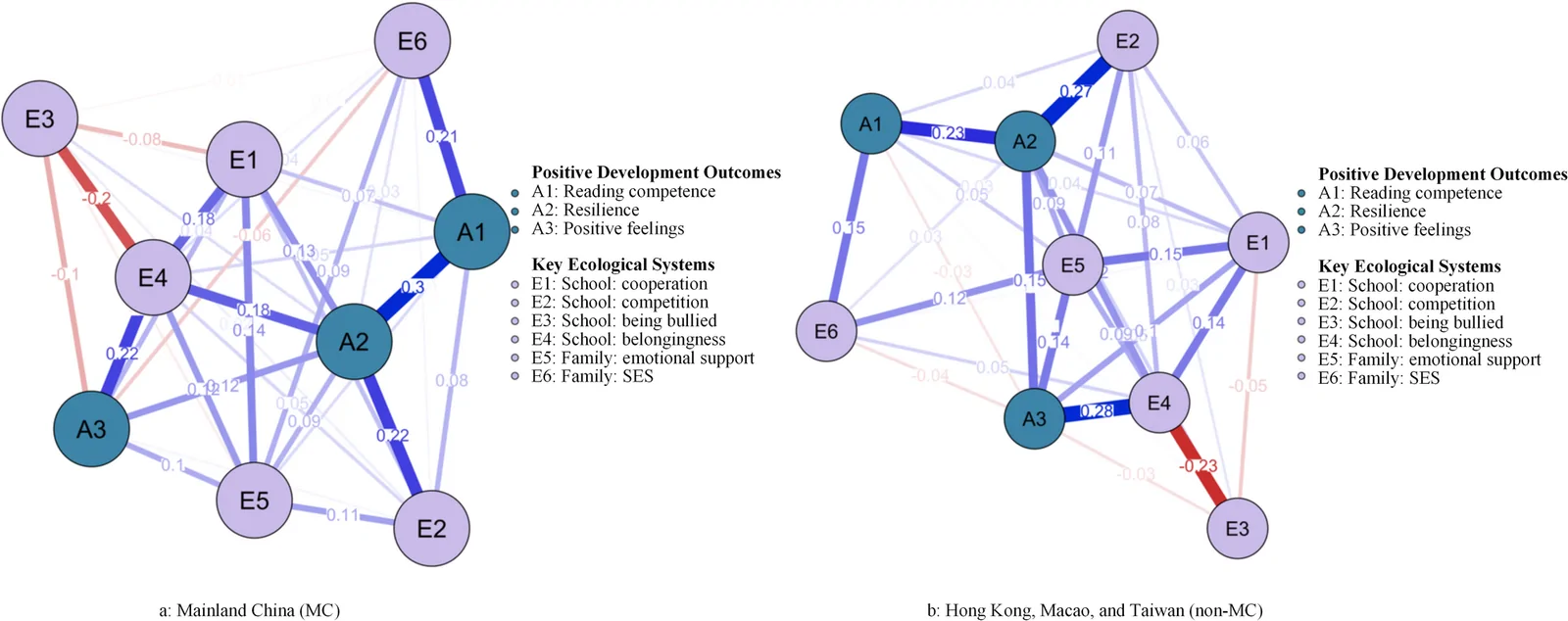
Networks of positive developmental outcomes and ecological factors in MC and non-MC adolescents. Panel (**a**) shows the network for adolescents from MC (*n* = 12,058). Panel (**b**) shows the network for adolescents from non-MC (*n* = 17,055), including Hong Kong, Macao, and Taiwan. Edges represent partial correlations, with blue and red indicating positive and negative associations, respectively; thicker edges denote stronger links. Blue nodes represent positive developmental outcomes: reading competence (A1), resilience (A2), and positive feelings (A3). Purple nodes represent ecological indicators—school system: cooperation (E1), competition (E2), being bullied (E3), and belongingness (E4); family system: emotional support (E5) and SES (E6). The Network Comparison Test indicated significantly higher global strength in the MC network (*p* = .014)

**Fig. 2:**
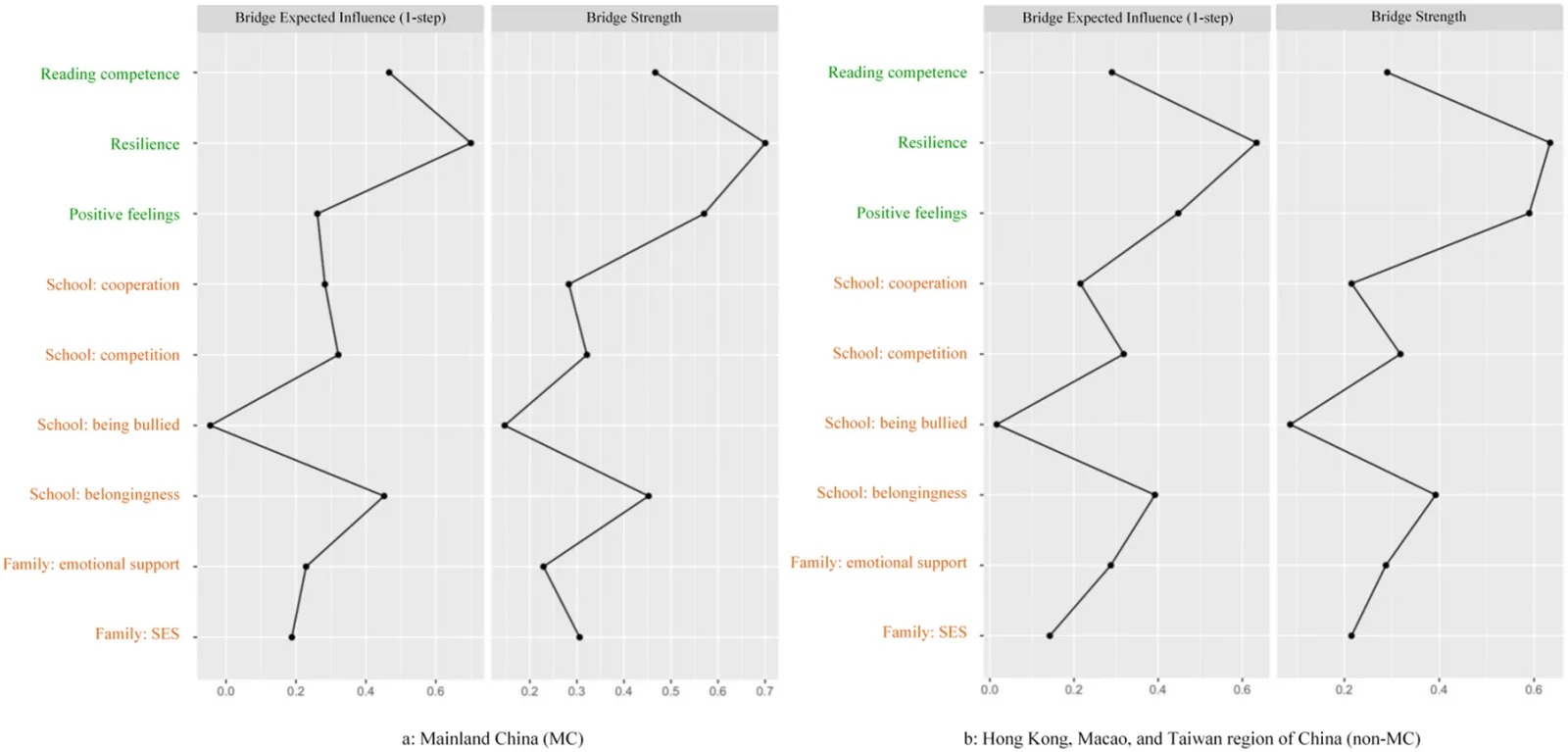
Bridge strength and bridge expected influence of positive developmental outcomes and ecological factors in MC and non-MC adolescents. Panel (**a**) shows the bridge expected influence and bridge strength for MC adolescents (*n* = 12,058). Panel (**b**) shows the corresponding indices for non-MC adolescents (*n* = 17,055), including adolescents from Hong Kong, Macao, and Taiwan. Higher bridge strength estimates indicate stronger connectivity between systems. Bridge expected influence reflects both the direction (positive or negative) and the magnitude of these cross-system connections

Network analysis revealed high stability in node and bridge estimates for both the MC and non-MC networks. Each network comprised six ecological system factors and three positive developmental outcomes. Among the 36 possible edges, 35 were statistically significant in the MC network and 33 were statistically significant in the non-MC network. The networks and their properties are presented in Fig. 1(a, b), Fig. 2(a, b), and Table 2. We summarize the key findings on network structure, global strength, bridge estimates, and edge differences below.

**Table 2:** Network estimates and comparison of network properties between MC and non-MC adolescents

Node name	Bridge strength			Bridge expected influence		
	MC est	non-MC est	*p*	MC est	non-MC est	*p*
Reading competence	0.47	0.29	0.001	0.47	0.29	0.001
Resilience	0.70	0.63	0.022	0.70	0.63	0.022
Positive feelings	0.57	0.59	0.501	0.26	0.45	0.001
School: cooperation	0.28	0.22	0.001	0.28	0.22	0.001
School: competition	0.32	0.32	0.885	0.32	0.32	0.885
School: being bullied	0.15	0.09	0.005	−0.05	0.02	0.001
School: belongingness	0.45	0.39	0.004	0.45	0.39	0.004
Family: emotional support	0.23	0.29	0.005	0.23	0.29	0.005
Family: SES	0.31	0.21	0.002	0.19	0.14	0.013

*Est* Estimates. Bridge expected influence refers to 1-step bridge expected influence. Higher scores of estimates indicate greater influence. *p* < .05 indicates a statistically significant difference between the two networks

The Network Comparison Test indicated significant differences in global network strength between MC and non-MC adolescents. Global strength was higher in the MC network (3.29) than in the non-MC network (3.10; *p* = .014). The network structure invariance test indicated significant differences between the MC and non-MC networks (*p* < .001), suggesting subcultural variation in the organization of ecological factors and positive development outcomes.

Notably, the bridge strengths of family SES, emotional support, being bullied, belongingness, school cooperation, and school competition with positive development were 0.31, 0.23, 0.45, 0.15, 0.32, and 0.28 for MC adolescents, and 0.21, 0.29, 0.39, 0.09, 0.32, and 0.22 for non-MC adolescents, respectively. Additionally, the bridge strengths of reading competence, resilience, and positive feelings with the ecological system factors were 0.47, 0.70, and 0.57 for MC adolescents, and 0.29, 0.63, and 0.59 for non-MC adolescents, respectively. The total bridge strength between ecological system factors and positive developmental outcomes was 1.74 for MC adolescents and 1.51 for non-MC adolescents. These findings suggest stronger overall cross-system connectivity between ecological system factors and positive development outcomes in the MC network.

Comparing edge weights between the MC and non-MC networks revealed several differences. The MC network showed weaker edge weights for the connections between positive feelings and belongingness (− 0.06, *p* < .001), positive feelings and emotional support (− 0.05, *p* < .001), positive feelings and being bullied (− 0.06, *p* < .001), and resilience and school competition (− 0.09, *p* = .002) compared with the non-MC network. Conversely, the MC network showed stronger edge weights for the connections between reading competence and resilience (0.07), resilience and school cooperation (0.05), reading competence and belongingness (0.05), resilience and belongingness (0.07), and reading competence and SES (0.07), with all *p*s < .001 after Bonferroni correction.

To examine whether missing-data handling influenced the network results, sensitivity analyses were conducted. Networks estimated using complete-case data yielded highly similar network structures, node and bridge patterns, and strongest edges (see Supplementary Fig. S1). Furthermore, Network Comparison Tests comparing participants with and without missing data showed no significant differences in the main network indices. In the MC sample, no significant differences were found in network structure (*p* = .999), global strength (*p* = .925), or edge weights (all *p*s > .815). In the non-MC sample, no significant differences were found in network structure (*p* = .999), global strength (*p* = .918), or node and bridge estimates (all *p*s > .646). These results suggest that missing data did not substantially influence the observed network patterns.

## Discussion

This study identified distinct ecological network structures of adolescents’ positive development in MC and non-MC contexts. Three main findings emerged. First, the MC network showed higher global strength than the non-MC network, indicating stronger overall connectivity among ecological factors and positive developmental outcomes. Second, reading competence and resilience showed stronger cross-system linkage roles in the MC network, whereas positive feelings showed higher bridge expected influence in the non-MC network. Third, several school-related variables showed stronger cross-system connections in the MC network, whereas family emotional support showed stronger cross-system connectivity in the non-MC network. Collectively, these findings suggest that adolescents’ positive development is structured through different family–school configurations across Chinese subcultural contexts. Given the large sample size and the modest magnitude of individual edge differences, the following interpretation gives priority to the overall patterns of global strength, bridge strength, bridge expected influence, and theoretically meaningful edge differences rather than isolated significant edges.

### Network tightness across MC and non-MC adolescents

The first key finding is that the MC network showed greater structural tightness than the non-MC network. In contrast, the non-MC network appeared less cohesive, suggesting relatively weaker cross-system connectivity and more differentiated family–school organization. Importantly, these contrasts point to qualitatively different ways of organizing adolescents’ developmental ecologies in MC and non-MC contexts, rather than indicating that one configuration is uniformly more desirable than the other. This difference supported Hypothesis 1, which predicted that subcultural differences would be reflected in network integration, operationalized as global network strength.

The difference in global strength can be interpreted through the degree of overlap between family and school systems. Ecological systems theory emphasizes that adolescents develop within interrelated contexts, and Epstein’s theory of overlapping spheres of influence conceptualizes family and school as partially overlapping spheres centered on students’ learning, development, and success [12]. This perspective has been widely used in research on school–family partnerships to examine how connections between families and schools are associated with family involvement, school satisfaction, and students’ academic outcomes [15, 18]. In the present study, this framework helps explain why the overall density of connections among family factors, school factors, and positive developmental outcomes may vary across different subcultural contexts.

In MC, the higher global strength may reflect a more integrated family–school ecology. In an examination-oriented educational context, academic achievement may function as a shared organizing goal for both families and schools. Family socioeconomic resources, parental emotional support, school belongingness, peer relations, cooperation, competition, and adolescents’ academic and adaptive resources may become more closely embedded within the same developmental ecology. The stronger global connectivity observed in the MC network therefore suggests that positive developmental outcomes are situated within a dense configuration of family and school factors. This interpretation helps explain the greater structural tightness of the MC network: family and school factors appear to be more closely coordinated around academic demands and adolescents’ adaptive resources.

In non-MC contexts, the lower global strength may reflect a more functionally differentiated organization of adolescents’ developmental ecology. Family support, school experiences, and positive developmental outcomes remain important, but their associations may be organized through more specific pathways. Prior studies suggest that Hong Kong and Macao show diversified educational values, more flexible forms of family involvement, and clearer functional boundaries between families and schools [23, 35]. In such contexts, families may play a more distinct role in supporting adolescents’ socioemotional well-being and broader developmental needs, while schools may retain a relatively distinct role in academic organization and educational authority. The lower global strength observed in the non-MC network can therefore be understood as reflecting a less tightly coupled family–school ecology, in which adolescents’ positive development is organized through more differentiated ecological pathways. The contrast in global strength suggests that subcultural variation within Greater China may be reflected in the structural organization of the adolescents’ ecological systems. The MC network appears to represent a more integrated family-school ecology, whereas the non-MC network seems to represent a more functionally differentiated ecology.

### Subcultural differences in the roles of positive developmental outcomes

Positive developmental outcomes occupied different structural positions in the MC and non-MC networks. In the MC network, reading competence showed a more central structural position, and resilience showed stronger cross-system linkage. In the non-MC network, positive feelings showed similar bridge strength but higher bridge expected influence than in the MC network, emerging, along with resilience, as one of the main cross-system linkage nodes. These patterns were broadly consistent with the MC-specific predictions of Hypothesis 2. However, because resilience showed weaker bridge strength and bridge expected influence in the non-MC network than in the MC network, Hypothesis 2 was only partially supported.

Across subcultural contexts, adolescents’ positive developmental outcomes occupied distinct structural positions within the ecological networks. In the MC region, reading competence plays a more central role in the network, with bridge strength and bridge expected influence significantly higher than those in the non-MC network. This pattern is consistent with the central status of academic achievement in the developmental ecology of MC [49]. This structural feature aligns with the exam-oriented educational system in MC, where reading comprehension is considered a fundamental threshold for academic success. Such an orientation may channel support from families, schools, and peers toward academic performance [28]. Furthermore, a cooperative school climate in MC may support student adaptation by helping students under academic pressure access support from others [50]. In contrast, in the non-MC network, positive feelings showed similar levels of bridge strength but significantly higher bridge expected influence than in the MC network, suggesting that emotional experiences play an important bridging role across systems. In the non-MC network, resilience and positive feelings occupy some of the most central bridging positions, with resilience showing the highest bridge strength overall. Thus, the positive feelings indicator functions as one of the core bridging nodes rather than a peripheral factor, although its influence is shared with other key indicators rather than dominating the entire network. This pattern may be partly attributed to the educational value orientations prevalent in the non-MC region, which place greater emphasis on psychological health and individual experiences. Emotional well-being, indexed in this study by positive feelings, may therefore represent a more salient developmental goal in non-MC contexts.

This study further revealed that resilience exhibited significantly higher bridge strength and bridge expected influence in the MC network compared to the non-MC network, suggesting its central role in cross-system coordination. This structural difference can be understood through the social ecology of resilience theory, which conceptualizes resilience as a culturally and contextually embedded adaptive resource shaped by the accessibility and integration of support within the ecological environment [46]. In the MC context, where family and school systems are highly interconnected, adolescents may be more likely to mobilize resources across multiple systems for self-regulation. In this context, resilience may serve as a pivotal bridge linking instrumental support, emotional support, and school climate [56]. In contrast, in the non-MC region, clearer boundaries between family and school systems, along with a greater emphasis on autonomy and systemic independence [24], may encourage adolescents to rely more on internal regulation within a single system—such as familial emotional support or peer relationships—rather than on cross-system integration to build resilience [35]. In the non-MC network, resilience showed relatively weaker cross-system connectivity, suggesting that its integrative role may be less pronounced in this context. Nevertheless, resilience continues to serve as an important asset for adolescents’ positive development in both contexts.

### Differential roles of school and family systems in MC and non-MC networks

The third key finding is that school and family systems showed different structural roles across MC and non-MC networks. In the MC network, school-related variables showed stronger cross-system connectivity and stronger connections with positive development than family-related variables, supporting the MC-specific prediction of Hypothesis 3. In contrast, in the non-MC network, family emotional support showed stronger cross-system connectivity than in the MC network; however, the family system as a whole did not show greater overall connectivity than the school system, so Hypothesis 3 received only partial support. These findings indicate structural differences in the relative roles of school and family systems in adolescents’ positive development across subcultural contexts.

In the MC network, the school system appeared to occupy a central structural position, with school-related variables showing stronger connections than family-related variables. Within the exam-oriented educational structure—particularly in the context of the College Entrance Examination (i.e., Gaokao)—schools may be closely connected with both students’ academic outcomes and psychological resources relevant to positive development [52]. Our findings are consistent with previous research in MC contexts demonstrating strong associations between school-related factors—such as school belongingness and academic competition—and key developmental indicators, including reading competence and resilience [58]. Notably, the educational ecology in MC is characterized by a distinctive coexistence of high academic competition and strong peer cooperation. A competitive school environment has been linked to more goal-directed behavior and stronger academic abilities, such as reading competence [49]. Cooperative climates are associated with peer support and belongingness, which may provide resources for emotion regulation and help alleviate stress [48]. These dynamics are consistent with a tightly integrated developmental ecology in which the school system occupies a structurally central role in adolescents’ positive development.

In the non-MC network, although school remains a central context in adolescents’ daily lives, emotional support within the family system shows strong connections with two key indicators of positive development: resilience and positive feelings. Prior research has linked emotional support with emotional comfort, lower loneliness and stress, and better psychological functioning [44]. Furthermore, emotional support has been linked to more adaptive emotion regulation skills, enabling adolescents to manage negative emotions more effectively [34], and to a greater capacity to form and maintain positive interpersonal relationships, thereby contributing to overall well-being [37]. These findings suggest that although the family system may not occupy a dominant structural position in adolescents’ developmental networks, emotional resources from the family remain important, particularly in the non-MC context.

School-related variables also showed differentiated roles within both MC and non-MC networks. School belongingness was the most central node in both networks, showing strong connections with indicators of positive development, as well as with cooperation, emotional support, and being bullied. Adolescents with a strong sense of school belongingness are more likely to perceive their environment as safe and supportive; such perceptions have been identified as protective factors in coping with stress and fostering psychological growth [36]. Cooperation and competition represent two important forms of social interaction within school settings. In the networks of this study, competition showed a stronger association with resilience than cooperation did. Although competition can increase psychological pressure, moderate and constructive competition may serve as a manageable challenge that supports adolescents’ development of coping skills. Being bullied has been identified as a risk factor that may undermine individual resources and disrupt support systems. This study identified a negative association between being bullied and school belongingness, suggesting that exclusion from peer groups undermines adolescents’ sense of connection at school. However, the impact of these risks may be buffered by the presence of protective factors, consistent with a risk-and-protective framework of development [38]. For instance, adolescents who perceive strong emotional support at home have been found to be less likely to experience emotional or behavioral problems over time [3]. Therefore, anti-bullying efforts should not only aim to reduce risks but also actively enhance protective resources to better support adolescent development.

### Strengths, limitations, and future directions

This study contributes to the literature by linking ecological systems theory with network analysis to examine subcultural variation in adolescents’ positive development. Theoretically, it examines the structural roles of family and school systems in adolescents’ positive development across two distinct subcultural contexts within Greater China—MC and non-MC regions. By integrating ecological systems theory with network analysis, the study clarifies the relative structural significance of school and family systems in each subcultural context and identifies key connecting variables for future empirical testing. This approach highlights ecosystemic differences in adolescent development within a shared cultural framework and contributes to emerging work in developmental psychology that foregrounds subcultural variation. Practically, the findings provide valuable insights for developing integrated support systems. Schools may need to monitor increasing academic pressures and strengthen resilience education. Parents may also enhance emotional support to complement school efforts in supporting both academic success and psychological well-being. Educational policies may also seek a better balance between academic demands and social-emotional learning to support adolescents’ holistic and positive development.

Despite these contributions, the findings should be interpreted with consideration of several limitations. First, this study relied on cross-sectional data from the PISA 2018 survey. The identified network structures provide valuable insights into subcultural differences at one time point, but they cannot capture changes in school and family systems over time. The sample was limited to 15-year-old students enrolled in school, and the MC sample was drawn from four Mainland Chinese regions available in PISA 2018. Thus, the findings should be interpreted as reflecting the available PISA-MC sample rather than all regions of Mainland China. PISA also uses a complex sampling design involving stratification, school-level clustering, and sampling weights. The present analyses used observed student-level data without sampling weights or school-level clustering adjustments, so the estimated networks should be interpreted as association structures within the analyzed samples rather than as fully population-representative estimates. Second, heterogeneity within the non-MC group should be considered. Although supplementary analyses showed broadly comparable global strength, bridge strength and bridge expected influence patterns across Hong Kong, Macao, and Taiwan, significant differences in overall network structure and several specific edges suggest that subregional variation may still exist. Most multi-item constructs showed broadly comparable measurement properties across MC and non-MC groups, but several measures showed indications of partial non-invariance. Cross-group network comparisons should be interpreted with caution. Third, this study concentrated on family and school systems and relied on adolescents’ self-reports. Future research should incorporate other ecological contexts, multi-informant reports from peers, parents, and teachers, and objective behavioral indicators to improve the reliability, ecological validity, and generalizability of the findings.

## Conclusion

This study used data from PISA 2018 and employed network analysis to compare the ecological association structures surrounding adolescents’ positive development in both MC and non-MC regions. The results indicated that the MC network showed stronger overall connectivity and greater structural integration among family factors, school factors, and positive developmental outcomes, suggesting a more cohesive family–school ecology. In contrast, the non-MC network appeared more loosely connected, suggesting greater functional differentiation among family factors, school factors, and positive developmental outcomes. Among the indicators of positive development, reading competence and resilience were more central in the MC network, with resilience also serving as a key bridging variable between ecological systems in both regions. In the non-MC network, positive feelings emerged as a core bridging indicator alongside resilience, although its role was shared with other key indicators rather than dominating the network. At the system level, school-related variables showed stronger associations with positive development in the MC network, indicating the structurally central position of school-related factors. In the non-MC network, emotional support from the family system was closely connected to resilience and positive feelings, suggesting that emotional resources are closely embedded in adolescents’ developmental networks. These findings reveal structural differences in adolescents’ developmental ecologies across Chinese subcultural contexts and offer empirical insights into how family and school systems are associated with adolescents’ positive development.

## Supplementary Information

Supplementary Material 1.

## Data Availability

The data that support the findings of this study are openly available in the PISA 2018 Database at https://www.oecd.org/en/data/datasets/pisa-2018-database.html.
