# Supplementary material for: Cultural comparison of positive development: network analyses of adolescents’ ecological systems in Mainland and non-Mainland China
Source: BMC Psychol. 2026 Jul 7;14:1407. doi: 10.1186/s40359-026-05106-z (PMC13621737; doi:10.1186/s40359-026-05106-z)
Supplement: Supplementary file 1 [file 40359_2026_5106_MOESM1_ESM.docx]

**Supplementary Materials**

**Supplementary Table S1** Intraclass correlation coefficients for study variables by sample

| **Variable** | **MC ICC** | **non-MC ICC** |
| --- | --- | --- |
| Reading competence | .086 | .052 |
| Resilience | .052 | .016 |
| Positive feelings | .012 | .018 |
| School: cooperation | .056 | .043 |
| School: competition | .019 | .029 |
| School: being bullied | .017 | .100 |
| School: belongingness | .054 | .061 |
| Family: emotional support | .042 | .037 |
| Family: SES | .449 | .278 |

*Note.* ICC = intraclass correlation coefficient. ICCs were estimated using random-intercept models with students nested within schools. Adjusted ICCs are reported because adjusted and unadjusted ICCs were identical.

**Supplementary Table S2** Missing data by variable and sample

| **Sample** | **Variable** | **Valid *n*** | **Missing *n*** | **Missing %** |
| --- | --- | --- | --- | --- |
| MC  (*N* = 12,058) | Age | 12,058 | 0 | 0.00 |
|  | Gender | 12,058 | 0 | 0.00 |
|  | Reading competence | 11,907 | 151 | 1.25 |
|  | Resilience | 11,982 | 76 | 0.63 |
|  | Positive feelings | 11,896 | 162 | 1.34 |
|  | School: cooperation | 11,916 | 142 | 1.18 |
|  | School: competition | 11,954 | 104 | 0.86 |
|  | School: being bullied | 11,899 | 159 | 1.32 |
|  | School: belongingness | 11,977 | 81 | 0.67 |
|  | Family: emotional support | 11,952 | 106 | 0.88 |
|  | Family: SES | 11,990 | 68 | 0.56 |
| non-MC  (*N* = 17,055) | Age | 17,055 | 0 | 0.00 |
|  | Gender | 17,055 | 0 | 0.00 |
|  | Reading competence | 16,599 | 456 | 2.67 |
|  | Resilience | 16,603 | 452 | 2.65 |
|  | Positive feelings | 16,500 | 555 | 3.25 |
|  | School: cooperation | 16,355 | 700 | 4.10 |
|  | School: competition | 16,601 | 454 | 2.66 |
|  | School: being bullied | 16,347 | 708 | 4.15 |
|  | School: belongingness | 16,601 | 454 | 2.66 |
|  | Family: emotional support | 16,406 | 649 | 3.81 |
|  | Family: SES | 16,777 | 278 | 1.63 |

*Note.* Missing % was calculated as (Total *N* − Valid *n*) / Total *N* × 100. The missing-rate ranges reported in the response letter refer to study variables only and therefore exclude region, age, and gender, which had no missing data.

**Supplementary Table S3** Comparisons between participants with complete data and those with missing data

| **Sample** | **Variable** | **Complete-data group *M* (*SD*)** | **Missing-data group *M* (*SD*)** | ***t*** | ***df*** | ***p*** | **Cohen’s *d*** |
| --- | --- | --- | --- | --- | --- | --- | --- |
| MC | Age | 15.774  (0.293) | 15.762  (0.296) | 0.80 | 440.99 | .424 | 0.040 |
|  | Gender | 1.520  (0.500) | 1.547  (0.498) | −1.09 | 441.87 | .279 | −0.054 |
|  | Reading competence | 0.084  (0.864) | −0.067  (0.911) | 2.65 | 271.66 | .009 | 0.170 |
|  | Resilience | −0.076  (0.953) | −0.055  (1.084) | −0.35 | 351.22 | .727 | −0.020 |
|  | Positive feelings | 0.103  (0.891) | 0.065  (0.938) | 0.64 | 259.81 | .524 | 0.042 |
|  | School: cooperation | 0.233  (1.007) | 0.183  (1.115) | 0.73 | 280.34 | .466 | 0.047 |
|  | School: competition | 0.422  (0.818) | 0.350  (0.842) | 1.48 | 323.64 | .141 | 0.086 |
|  | School: being bullied | −0.235  (0.877) | −0.141  (1.002) | −1.49 | 261.54 | .137 | −0.100 |
|  | School: belongingness | −0.145  (0.907) | −0.199  (0.948) | 1.01 | 348.48 | .312 | 0.058 |
|  | Family: emotional support | 0.006  (0.931) | −0.023  (0.932) | 0.55 | 322.31 | .585 | 0.032 |
|  | Family: SES | −0.354  (1.086) | −0.643  (1.081) | 4.89 | 364.87 | < .001 | 0.267 |
| non-MC | Age | 15.781  (0.296) | 15.749  (0.289) | 3.67 | 1420.36 | < .001 | 0.108 |
|  | Gender | 1.498  (0.500) | 1.597  (0.491) | −6.77 | 1418.41 | < .001 | −0.200 |
|  | Reading competence | −0.317  (0.924) | −0.460  (1.007) | 3.86 | 823.79 | < .001 | 0.149 |
|  | Resilience | −0.264  (0.895) | −0.288  (1.031) | 0.63 | 821.70 | .530 | 0.025 |
|  | Positive feelings | 0.019  (0.993) | −0.150  (1.080) | 3.95 | 709.57 | < .001 | 0.163 |
|  | School: cooperation | 0.154  (0.969) | 0.033  (1.090) | 2.50 | 544.07 | .013 | 0.117 |
|  | School: competition | 0.219  (0.839) | 0.055  (0.896) | 4.96 | 828.83 | < .001 | 0.189 |
|  | School: being bullied | −0.095  (0.968) | 0.030  (1.131) | −2.46 | 533.28 | .014 | −0.118 |
|  | School: belongingness | −0.245  (0.812) | −0.380  (0.791) | 4.60 | 842.33 | < .001 | 0.168 |
|  | Family: emotional support | −0.267  (0.910) | −0.430  (0.949) | 4.03 | 606.15 | < .001 | 0.175 |
|  | Family: SES | −0.438  (0.956) | −0.479  (1.034) | 1.20 | 1036.51 | .231 | 0.042 |

*Note.* Complete-data group = participants with no missing data on the variables included in the missingness check; Missing-data group = participants with any missing data. Cohen’s *d* > 0 indicates that the complete-data group had a higher mean than the missing-data group. Gender was coded as in the source data.

**Supplementary Table S4** Measurement invariance tests for multi-item constructs across MC and non-MC groups

| **Construct** | **Configural model** | | | | **Metric model** | | | | **Change in fit and χ² difference** | | | |
| --- | --- | --- | --- | --- | --- | --- | --- | --- | --- | --- | --- | --- |
|  | CFI | TLI | RMSEA | SRMR | CFI | TLI | RMSEA | SRMR | ΔCFI | ΔRMSEA | ΔSRMR | Δχ² *p* |
| Reading competence | 1.000 | 1.000 | .000 | .000 | .991 | .974 | .093 | .032 | −.009 | .093 | .032 | < .001 |
| Resilience | .979 | .959 | .075 | .020 | .979 | .970 | .064 | .021 | .000 | −.011 | .001 | .041 |
| Positive feelings | 1.000 | 1.000 | .000 | .000 | 0.989 | .966 | .119 | .039 | −.011 | .119 | .039 | < .001 |
| School: belongingness | .852 | .753 | .182 | .073 | 0.846 | .799 | .164 | .078 | −.006 | −.018 | .004 | < .001 |
| School: being bullied | 1.000 | 1.000 | .000 | .000 | 0.979 | .938 | .124 | .046 | −.021 | .124 | .046 | < .001 |
| School: competition | 1.000 | 1.000 | .000 | .000 | 0.999 | .996 | .044 | .014 | −.001 | .044 | .014 | < .001 |
| School: cooperation | 1.000 | 1.000 | .000 | .000 | 1.000 | .999 | .022 | .006 | .000 | .022 | .006 | < .001 |
| Family: emotional support | 1.000 | 1.000 | .000 | .000 | 1.000 | .999 | .027 | .009 | .000 | .027 | .009 | < .001 |

*Note.* CFI = comparative fit index; TLI = Tucker–Lewis index; RMSEA = root mean square error of approximation; SRMR = standardized root mean square residual. Metric models constrained factor loadings to be equal across groups. Δ values represent metric model fit minus configural model fit. χ² difference *p* values are reported for metric versus configural models.

**Supplementary Table S5** Descriptive statistics for each non-MC subregion

| **Variable** | | **Statistic** | **Hong Kong** | **Macao** | **Taiwan** |
| --- | --- | --- | --- | --- | --- |
| Age | | *n* | 6,037 | 3,775 | 7,243 |
|  |  | *M* (*SD*) | 15.73 (0.29) | 15.83 (0.29) | 15.79 (0.30) |
| Gender | girls | *n* (%) | 2,955 (48.95%) | 1,862 (49.32%) | 3,624 (50.03%) |
|  | boys | *n* (%) | 3,082 (51.05%) | 1,913 (50.68%) | 3,619 (49.97%) |
| Reading competence | | *n* | 5,758 | 3,745 | 7,096 |
|  |  | *M* (*SD*) | −0.22 (0.92) | −0.42 (0.84) | −0.36 (0.97) |
| Resilience | | *n* | 5,702 | 3,765 | 7,136 |
|  |  | *M* (*SD*) | −0.29 (0.91) | −0.34 (0.83) | −0.21 (0.93) |
| Positive feelings | | *n* | 5,680 | 3,747 | 7,073 |
|  |  | *M* (*SD*) | −0.06 (0.94) | −0.08 (1.04) | 0.12 (1.01) |
| School: cooperation | | *n* | 5,568 | 3,741 | 7,046 |
|  |  | *M* (*SD*) | 0.07 (0.93) | 0.04 (0.93) | 0.27 (1.01) |
| School: competition | | *n* | 5,718 | 3,766 | 7,117 |
|  |  | *M* (*SD*) | 0.13 (0.83) | 0.14 (0.82) | 0.32 (0.85) |
| School: being bullied | | *n* | 5,571 | 3,741 | 7,035 |
|  |  | *M* (*SD*) | 0.11 (1.05) | 0.14 (1.03) | −0.37 (0.79) |
| School: belongingness | | *n* | 5,700 | 3,765 | 7,136 |
|  |  | *M* (*SD*) | −0.39 (0.70) | −0.40 (0.68) | −0.06 (0.91) |
| Family: emotional support | | *n* | 5,589 | 3,754 | 7,063 |
|  |  | *M* (*SD*) | −0.36 (0.88) | −0.34 (0.92) | −0.17 (0.92) |
| Family: SES | | *n* | 5,839 | 3,766 | 7,172 |
|  |  | *M* (*SD*) | −0.52 (1.02) | −0.52 (0.91) | −0.33 (0.92) |

*Note.* For age and study variables, *n* and *M* (*SD*) are reported. For gender, *n* (%) is reported. Except for age and gender, the values are based on standardized scores. Hong Kong, Macao, and Taiwan are presented separately to examine heterogeneity within the non-MC group.

**Supplementary Table S6** Pairwise Network Comparison Tests among Hong Kong, Macao, and Taiwan

| **Panel A. Network structure and global strength invariance tests** | | | | | | | |
| --- | --- | --- | --- | --- | --- | --- | --- |
| **Pairwise comparison** | **Network *M*** | **Network *p*** | **Global strength 1** | | **Global strength 2** | ***S*** | **Global strength *p*** |
| Hong Kong vs. Macao | 0.076 | .038 | 2.914 | | 3.098 | 0.184 | .605 |
| Hong Kong vs. Taiwan | 0.079 | .007 | 2.914 | | 3.147 | 0.233 | .088 |
| Macao vs. Taiwan | 0.085 | .005 | 3.098 | | 3.147 | 0.049 | .814 |
|  | | | | | | | |
| **Panel B. Bridge strength invariance tests by node** | | | | | | | |
| **Pairwise comparison** | **Node** | | | **Bridge strength difference** | | | ***p*** |
| Hong Kong vs. Macao | Reading competence | | | −0.046 | | | .463 |
|  | Resilience | | | −0.022 | | | .713 |
|  | Positive feelings | | | 0.020 | | | .765 |
|  | School: cooperation | | | 0.070 | | | .055 |
|  | School: competition | | | −0.047 | | | .182 |
|  | School: being bullied | | | −0.043 | | | .571 |
|  | School: belongingness | | | 0.001 | | | .969 |
|  | Family: emotional support | | | −0.034 | | | .336 |
|  | Family: SES | | | 0.004 | | | .932 |
| Hong Kong vs. Taiwan | Reading competence | | | −0.081 | | | .061 |
|  | Resilience | | | 0.076 | | | .093 |
|  | Positive feelings | | | −0.049 | | | .220 |
|  | School: cooperation | | | 0.041 | | | .186 |
|  | School: competition | | | 0.001 | | | .957 |
|  | School: being bullied | | | −0.056 | | | .111 |
|  | School: belongingness | | | 0.023 | | | .391 |
|  | Family: emotional support | | | −0.006 | | | .839 |
|  | Family: SES | | | −0.057 | | | .090 |
| Macao  vs. Taiwan | Reading competence | | | −0.035 | | | .447 |
|  | Resilience | | | 0.099 | | | .085 |
|  | Positive feelings | | | −0.068 | | | .228 |
|  | School: cooperation | | | −0.030 | | | .414 |
|  | School: competition | | | 0.049 | | | .111 |
|  | School: being bullied | | | −0.013 | | | .743 |
|  | School: belongingness | | | 0.022 | | | .501 |
|  | Family: emotional support | | | 0.028 | | | .398 |
|  | Family: SES | | | −0.060 | | | .170 |

| **Panel C. Significant edge invariance tests (*p* < 0.05)** | | | | |
| --- | --- | --- | --- | --- |
| **Pairwise comparison** | **Node 1** | **Node 2** | **Edge difference statistic** | ***p*** |
| Hong Kong vs. Macao | Resilience | School: cooperation | 0.060 | .013 |
|  | Reading competence | School: being bullied | 0.048 | .014 |
|  | School: cooperation | School: being bullied | 0.076 | .001 |
|  | School: being bullied | School: belongingness | 0.071 | .004 |
|  | School: competition | Family: emotional support | 0.047 | .045 |
|  | School: being bullied | Family: emotional support | 0.052 | .038 |
|  | School: being bullied | Family: SES | 0.057 | .004 |
|  | School: belongingness | Family: SES | 0.047 | .025 |
| Hong Kong vs. Taiwan | Resilience | School: cooperation | 0.043 | .035 |
|  | Resilience | School: belongingness | 0.046 | .031 |
|  | School: competition | School: belongingness | 0.047 | .017 |
|  | School: being bullied | Family: emotional support | 0.038 | .045 |
|  | Reading competence | Family: SES | 0.079 | .001 |
|  | School: cooperation | Family: SES | 0.031 | .021 |
| Macao  vs. Taiwan | Reading competence | Resilience | 0.068 | .007 |
|  | Reading competence | School: being bullied | 0.068 | .001 |
|  | Positive feelings | School: being bullied | 0.048 | .024 |
|  | School: cooperation | School: being bullied | 0.044 | .046 |
|  | School: competition | School: belongingness | 0.051 | .023 |
|  | School: being bullied | School: belongingness | 0.040 | .048 |
|  | School: competition | Family: emotional support | 0.044 | .048 |
|  | Reading competence | Family: SES | 0.085 | .001 |
|  | Resilience | Family: SES | 0.047 | .011 |
|  | School: being bullied | Family: SES | 0.057 | .001 |

*Note.* Network *M* = test statistic for the network invariance test; *S* = test statistic for the global strength invariance test. Global strength 1 and global strength 2 correspond to the first and second regions listed in each pairwise comparison, respectively. Bridge strength difference was calculated as the first region minus the second region in each pairwise comparison. Panel C lists edge invariance tests with *p* < .05. All *p* values are uncorrected, as returned by the NetworkComparisonTest output. Variable labels were harmonized with the manuscript terminology.

**Supplementary Fig. S1.** Complete-case networks of positive development indicators and ecological factors in MC and non-MC adolescents

*Note*. Panel (a) shows the complete-case network for MC adolescents. Panel (b) shows the complete-case network for non-MC adolescents, including adolescents from Hong Kong, Macao, and Taiwan. Edges represent partial correlations, with blue and red edges indicating positive and negative associations, respectively; thicker edges indicate stronger associations. Blue nodes represent positive development indicators: reading competence (A1), resilience (A2), and positive feelings (A3). Purple nodes represent ecological factors, including school system factors: cooperation (E1), competition (E2), being bullied (E3), and belongingness (E4); and family system factors: emotional support (E5) and socioeconomic status (SES; E6).
